# Functional outcomes in bilateral upper limb Amelia patient with scoliosis post vertical expandable prosthetic titanium rib (VEPTR) application: A case report

**DOI:** 10.1016/j.ijscr.2020.03.007

**Published:** 2020-03-07

**Authors:** Abdullah AlMarshad, Ibrahim AlMazrua, Rakan Al-Haidey, Zayed AlZayed

**Affiliations:** King Faisal Specialist Hospital and Research Centre, Saudi Arabia

**Keywords:** Phocomelia, Amelia, Rare, Congenital, Scoliosis

## Abstract

**Introduction:**

Congenital upper limb Amelia is one of the extremely rare conditions in the world. Defined as complete absence of a limb which may present as isolated defect or as a part of syndrome with associated anomalies.

**Presentation of the case:**

We report a case of a medically free 6-year-old boy with bilateral upper limb Amelia associated with right thoracolumbar idiopathic Scoliosis.

**Discussion:**

Treatment for early onset scoliosis includes either posterior spinal fusion and instrumentation, or Vertical Expandable Prosthetic Titanium Rib (VEPTR). The choice of care for our patient was decided to be VEPTR alone as definitive management. Up to our knowledge, there are very scanty articles published regarding treatment for such cases.

**Conclusion:**

Patient underwent vertical expandable prosthetic titanium rib (VEPTR) application for his large 45-degree curve as a definitive treatment and still have his preoperative physical functions, in terms of using lower limbs in daily living activities.

## Highlights

• Amelia has been associated with idiopathic scoliosis in non-syndromic patients.
• VEPTR device use in management was chosen as a definitive treatment in order to preserve spinal range of motion.
• Up to our knowledge there is no consensus on the definitive treatment of scoliosis with congenital limb agenesis patients.

## 1 Introduction

Amelia can be defined as a congenital complete absence of one or more limbs. It can present as isolated defect or with associated anomalies, including but not limited to cleft lip or palate, neural tube defects, diaphragmatic defects, absent kidneys, head malformation and scoliosis [[1], [2], [3], [4]].

The exact pathogenesis is still unclear, but mostly it is believed that it occurs as a sporadic event [5]. Moreover, some researchers found that it may be related to some teratogens such as thalidomide, alcohol, maternal diabetes and vascular compromise by amniotic bands [[6], [7], [8]]. The prevalence of this rare condition among 23.1 million births from 1968 to 2006 was found to be 1.41 per 100,000 births, ranging from 0.42 to a maximum of 2.44 [9].

Furthermore, when we try to describe the relationship between Amelia and scoliosis, it becomes more difficult due to the rarity of the condition and the scanty research done on this topic, which in turn stimulated us to report this case of bilateral upper limb Amelia and scoliosis. The work has been reported in line with the SCARE criteria [10]. This case was reported in accordance with the SCARE Guidelines [10].

## 2 Presentation of case

The mother is 43 years old and the father is 49 years old, both healthy non consanguineous parents who live in a village. No known teratogenic exposure during pregnancy. They have eight healthy children, six boys and two girls except our case who is the last child for the family. The child is 6 years old medically free, product of normal spontaneous vaginal delivery at full term without any intensive care unit admission. No reports available for his status during delivery and no ultrasound report. According to the mother his weight at delivery was 1.9 kg.

**Fig. 1:**
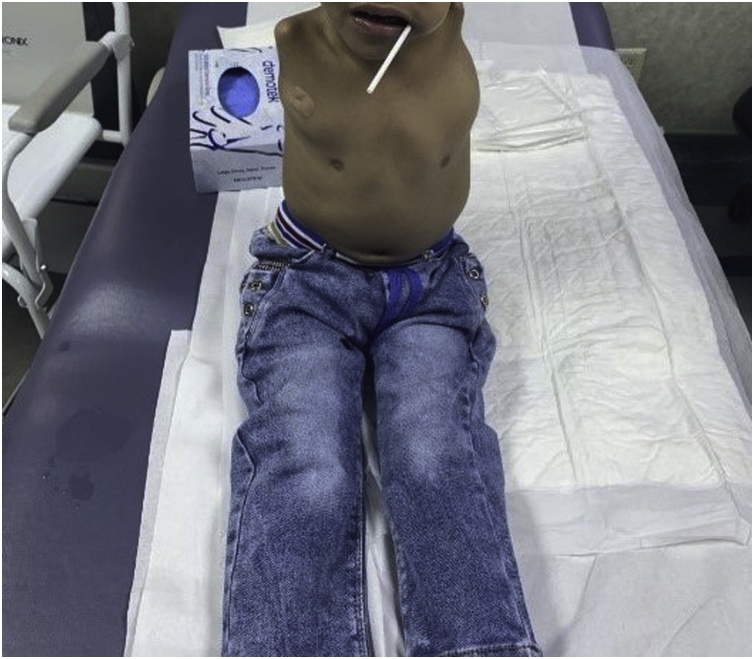
Upon examination, no dysmorphic features where noted aside from bilateral absent upper limbs from the shoulder.

**Fig. 2:**
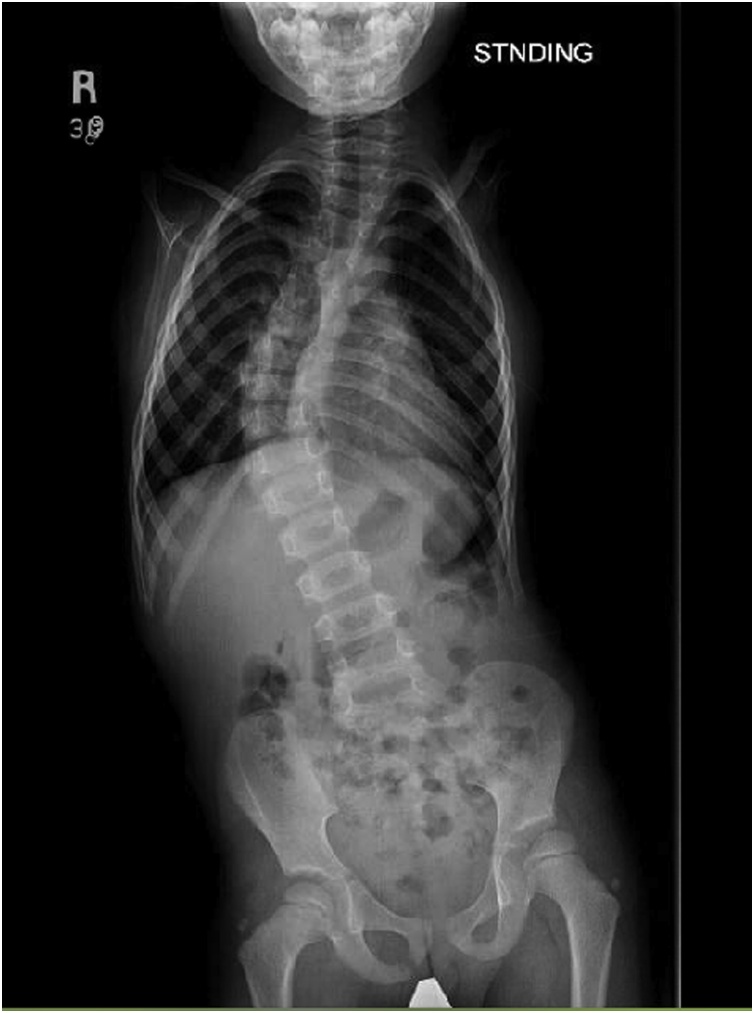
Right thoracolumbar curve of 45 degrees was identified in imaging without any other vertebral or cord abnormalities in MRI.

First time seen in our clinic at 23 months of age where he was referred to our genetic department in the hospital. They found that he has SNP (single nucleotide polymorphism) positive duplication in chromosome y or x. Same mutation was found in the father. The child started to sit at 9 months and walk at 18 months. The parents still didn’t enroll him in any school in the village. Upon examination, no dysmorphic features where noted aside from bilateral absent upper limbs from the shoulder (Fig. 1). He has a right limb leg length discrepancy of 2 cm with pelvic tilt with a positive Adams forward bending test with right rib hump. Normal distal neurovascular examination. He has about 90 degrees of flexion and 20–30 degree of extension and lateral bending upon range of motion examination for the back. Abdominal Ultrasound done in our hospital showed no abnormalities. Right thoracolumbar curve of 45 degrees was identified in imaging without any other vertebral or cord abnormalities in MRI (Fig. 2). Bone mineral density exam was normal. The family was counseled about the child’s condition and the options for treatment. They agreed for growing rod method. In March 2019 he underwent VEPTR application.

**Fig. 3:**
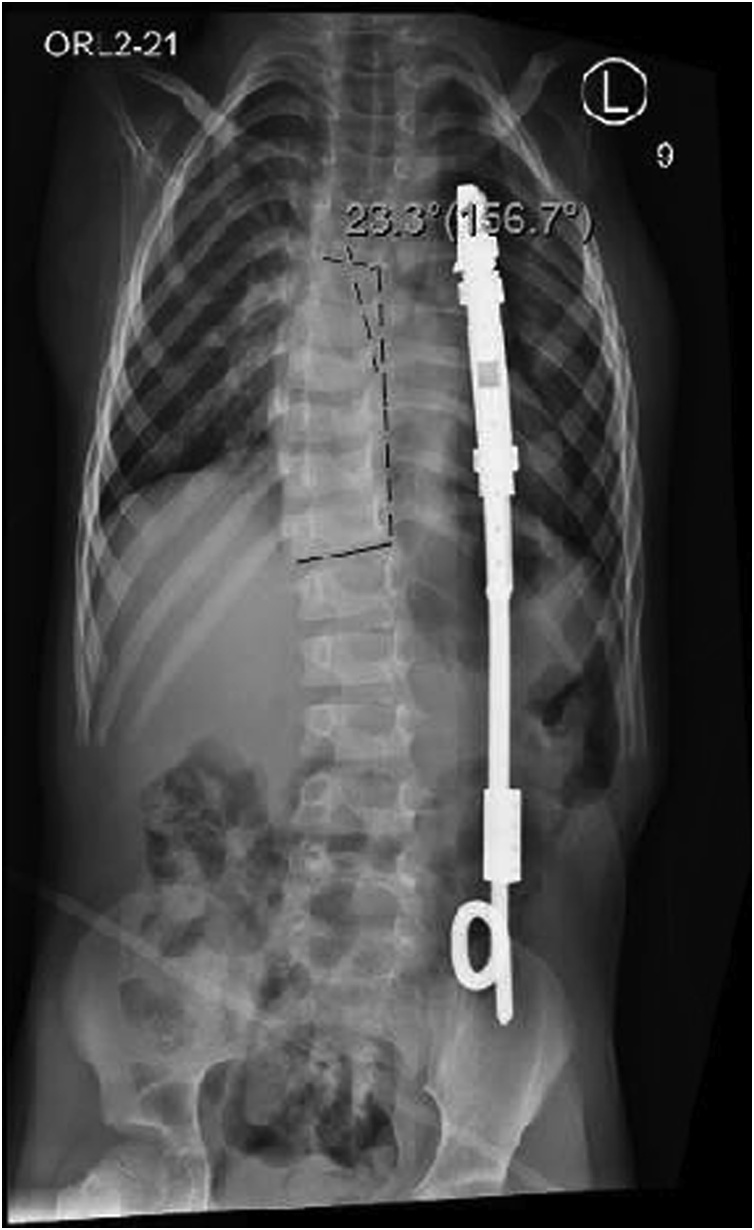
Reduction of 18 degrees was achieved for the thoracolumbar curve.

Our aim was to keep it as the definitive fixation with periodic lengthening, as well as to preserve spine range of motion as much as possible to maintain his independence in daily living activities by the use of his lower limbs. Rib to ilium VEPTR applied from the 5th and 6th rib to iliac bone with cradle and ala hook and multiple anchor connections. Reduction of 18 degrees was achieved for the thoracolumbar curve (Fig. 3).

**Figs. 4 and 5:**
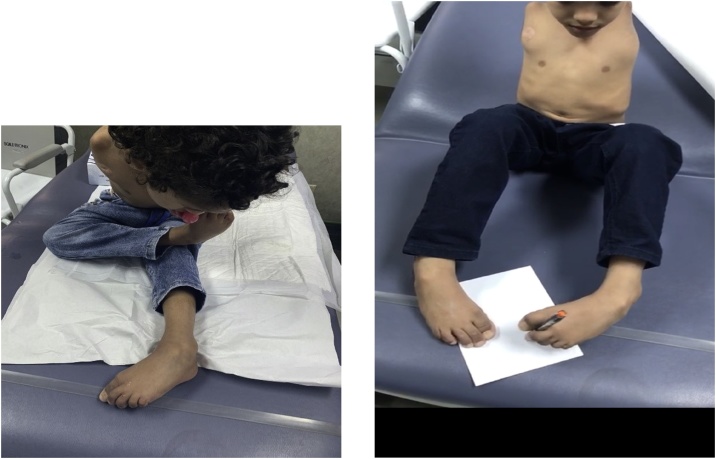
Maintaining preoperative range of motion for the spine and able to use his lower limbs for feeding, drawing and combing his hair.

Post-surgical intervention the child was seen in the clinic. Maintaining preoperative range of motion for the spine and able to use his lower limbs for feeding, drawing and combing his hair (Figs. 4 and 5). Patient followed up for 1 years and will be followed up until the skeletal maturity.

## 3 Discussion

The presentation of Amelia is a congenitally absent limb. This has been associated with early onset scoliosis, as mentioned by Olgun ZD et al. [11]. They reported 4 cases where patients had either amelia or severe phocomelia with idiopathic scoliosis, with the apex of the curvature pointing towards the absent limb. They postulated that their patient’s scoliosis was due to posture correction as well as balance, as they had no vertebral malformations upon further investigation.

To add upon this, Powers et al. [12] performed a retrospective study to determine the incidence of scoliosis in patients with upper limb skeletal abnormalities, and concluded that patients with Amelia had a higher incidence of idiopathic scoliosis. (Unilateral Amelia = 50% incidence/bilateral = 100%) [12].

Treatment for early onset scoliosis includes either posterior spinal fusion and instrumentation, or Vertical Expandable Prosthetic Titanium Rib (VEPTR). In his paper, Studer, D et al. [13] stated that the decision to undergo fusion surgery post expansion treatment is determined by curve progression, operative complications and failure to distract from initial management. Magnitude of the deformity was found to be the most definitive indication to progress to spinal fusion. His patients were divided into 3 groups; VEPTR graduates without fusion therapy, VEPTR with final fusion surgery and removal of VEPTR without final fusion. In this cohort, only 30% of the patients with congenital scoliosis required final fusion surgery.

Moreover, the group with final fusion had decrease in spinal range of motion compared to other groups. On the other hand, in our case there was no change in the activities before and after VEPTR device as definitive treatment.

In regards to VEPTR outcomes, Gantner, A. S. et al. [14] performed a retrospective case series consisting of 32 children with spinal deformities managed with expansion therapy with radiological and clinical data pre/post op with a concurrent follow up biennially. This study postulated that decrease in correction potential in VEPTR treatment at an average deadline of 5.5 years might be due to the ossifications around the implant which may either stiffen the spine or thorax [14]. This theory has been aided by the fact that VEPTR is maintained by a two-point fixation, where Lattig F et al. [15] found that constant movement and migration introduced new bone growth around the implant [15]. This theory supporting the option of treatment have been chosen in our case, to avoid the fusion and concurrent stiffness in the spinal range of motion and to keep the functions in addition to prevent the curve progression.

Sanker WN et al. [16] found a phenomenon where VEPTR implant might increase likelihood of periosteal injury upon placement, yielding decreased correction due to spinal autofusion [16]. In our case the correction was 18 degrees’ reduction in thoracolumbar curve which is considered acceptable as our target is to maintain the spinal Range of motion.

Robert F. Murphy et al. [17] completed a study where the use of VEPTR without fused ribs was compared to its habitual method of VEPTR with fused ribs. He stated upon the former that from insertion to the final follow up, they were able to control correction in the coronal plane by showing a 24% improvement in the Cobb angle [16]. In contrast to this, Dayer R et al. [18] showed a preference of VEPTR to be used only in two classes of patients: those with congenital scoliosis associated with rib fusion, and spinal deformities found in non-ambulating myelodysplasia patients [18].

R.M. Holewijn et al. [19] in their study titled Spinal fusion limits upper body range of motion during gait without inducing compensatory mechanisms in adolescent idiopathic scoliosis patients concluded that the thoracic spine range of motion is significantly decreased after spinal fusion during walking in addition to the pelvis Range of motion [19]. This issue was tackled in our case by using VEPTR device.

The choice of care for our patient was decided to be VEPTR alone as definitive management. Up to our knowledge, there are very scanty articles published regarding treatment for such cases.

## 4 Conclusion

Amelia is one of the very rare and challenging conditions affecting limb development in the world. Progressive scoliosis in these patients should be identified and treated early to prevent cardiopulmonary complications as well as to preserve the ability to do daily living activities. The use of VEPTR instead of fusion can be of great importance to preserve as much as functional abilities. To our knowledge Very scanty research done in this field. We recommend more researches to strengthen our experience.

## Declaration of Competing Interest

None.

## Source of funding

None.

## Ethical approval

The study was approved by institutional review board by king faisal specialist hospital and research centre.

## Consent

Written informed consent was obtained from the patient’s parents for publication of this case report and accompanying images. A copy of the written consent is available for review by the editor-in-chief of this journal on request.

## Author contribution

Abdullah Almarshad contributes the paper with writing the paper, data collection and data analysis.

Ibrahim AlMazrua contributes the paper with writing the paper, data collection, data analysis and interpretation.

Rakan Al-Haidey contributes the paper with data collection and data analysis.

Zayed AlZayed contributes the paper with data analysis, interpretation and whole management.

## Registration of research studies

Not required.

## Guarantor

Abdullah Almarshad.

## Provenance and peer review

Not commissioned, externally peer-reviewed.
